# In vitro Comparison of the Accuracy (Precision and Trueness) of Seven Dental Scanners 

**DOI:** 10.30476/DENTJODS.2020.83485.1047

**Published:** 2021-03

**Authors:** Fariborz Vafaee, Farnaz Firouz, Mahsa Mohajeri, Reza Hashemi, Somayeh Ghorbani Gholiabad

**Affiliations:** 1 Dental Implant Research Center, Dept. of Prosthodontics, Dental Faculty, Hamedan University of Medical Sciences, Hamedan, Iran; 2 Dept. of Prosthodontics, Dental Faculty, Hamedan University of Medical Sciences, Hamedan, Iran; 3 Post Graduate Student, Dept. of Prosthodontics, Dental Faculty, Hamedan University of Medical Sciences, Hamedan, Iran; 4 Dept. of Prosthodontics, Dental Faculty, Yasuj University of Medical Sciences, Yasuj, Iran.; 5 Dept. of Biostatistics, School of Public Health, Hamedan University of Medical Sciences, Hamedan, Iran

**Keywords:** Dental Scanners, Precision, Trueness

## Abstract

**Statement of the Problem:**

The mechanisms of operation of dental scanners are based on different technologies. Considering these differences, there are many types of scanners available in the market.

**Purpose:**

This in vitro study aimed to compare the accuracy (precision and trueness) of seven commonly used dental scanners.

**Materials and Method:**

In this in vitro experimental study, the accuracy of 7 common extra oral scanners (Sirona ineos inLab, Sirona X5, Dentium, Imes Icore 350I, Amann Girrbach, 3shape D700, and 3Shape E3) were evaluated. Each of scanners performed 7 scans of implant abutment of SIC (SIC MAX.GH1). Data from each scanner were then compared to data received from 3Shape Trios intra oral scanner as a reference. For evaluating the accuracy of each scanner, trueness and precision was evaluated. Collected data were analyzed using Kruskal Wallis and Bonferroni tests via SPSS version 22.

**Results:**

Descriptive statistics showed the best trueness was for 3Shape E3 scanner with the average of 35.37µm and the worst trueness belonged to Sirona x5 scanner with the average of 51.75µm. Furthermore, the best precision was achieved for 3Shape E3 scanner with the average of 35.34, while the lowest precision was detected in 3Shape D700. The scanners had statistically significant differences with each other in terms of trueness and precision (*p*<0.05).

**Conclusion:**

Based on the results of this study, the extra oral scanner, 3shape E3, had the best trueness and precision. The lowest amount of trueness among the studied scanners was for the extra oral scanner, Sirona x5, and the lowest precision was for scanner 3shape D700.

## Introduction

Computer-aided design and computer-aided manufacturing (CAD/CAM) technology was introduced to dentistry in the 1980 [ 1 - 2 ]. Currently, this technology is being exceedingly crowd-pleasing due to fewer clinical sessions leading to patient comfort and it is routinely used for designing and fabrication of fixed partial dentures, removable partial denture frameworks, maxillofacial prostheses, and complete dentures [ 3 ]. Processing chain of CAD/CAM technology consists of three different steps including surface scanning, designing of restoration, and manufacturing of the prosthesis, which can be milled or 3D printed using different types of materials [ 1 , 4 ]. The first step is achieved by using dental scanners, which are available in two main groups of extra oral and intra oral scanners [ 1 , 5 ]. 

Intraoral scanners use active triangulation, confocal microscopy or wave front sampling principles to obtain a digital scan of the patient’s dental arch chairside by use of camera image impression or video image impression technology [ 3 , 6 - 7 ]. Extraoral scanners provide scans of casts in a laboratory using laser, structured light, or contact technology. Optical scanners (laser and structured light) are not influenced by density of the scanned object [ 1 , 8 ] and are faster than contact scanners [ 1 , 9 ]. However, they may be affected by surface properties of the object such as its shine and glossiness. Surface density may affect the accuracy of contact scanners; however, their precision is not influenced by the optical characteristics of the object [ 1 , 10 ]. 

Enhancement in accuracy is one of the most important aims of digital dentistry and computer aided technology can decrease the discrepancies that occur during conventional method of impression making and crown fabrication. According to different technologies of intra and extra oral scanners, different companies release different types of scanners. There is inconsistent information about the accuracy of crowns made with these technologies, considering precision and trueness as the two important factors determining the accuracy [ 11 ]. Precision is defined as the closeness of the measurements made by each scan compared with the same measurements made by the previous/next scan of the same scanner. Thus, a high-precision scan has higher reproducibility. Trueness describes the difference between the scanned dimensions and the actual dimensions of an object. Thus, a scanner with high trueness is capable of capturing scans closer to the actual dimensions of an object [ 11 - 12 ]. 

Studies on the accuracy of restorations fabricated on implant abutments by the CAD/CAM technology are limited and they have reported controversial results [ 2 , 13 ]. For instance, Hack and Patzelt [ 14 ] found that 3Shape Trios intraoral scanner had the highest trueness. 

Jeong *et al*. [ 15 ] found that the precision of intraoral scanners decreases as their distance from the object in creases. De Villaumbrosia *et al*. [ 1 ] reported that Zeno Scan extraoral scanner had the highest accuracy and precision. 

Considering the existing literature, comparison of the accuracy of different scanners and reaching a conclusion regarding their precision and the most efficient scanner for use in the laboratory and clinical setting would be difficult. Thus, this *in vitro* study aimed to assess and compare the precision and trueness of seven commonly used extra oral scanners. 

## Materials and Method

**Figure 1:**
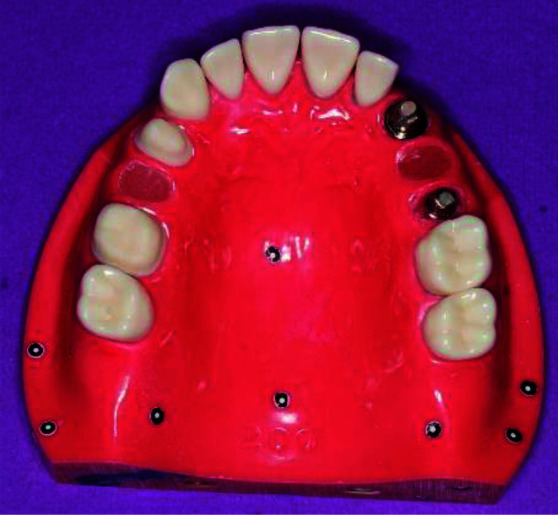
Implant abutment mounted in a typodont (the original model for scanning)

In this *in vitro* experimental study, the accuracy of seven common extra oral scanners (Sirona ineos inLab, Sirona X5, Dentium, Imes Icore 350I, Amann Girrbach, 3Shape D700, and 3Shape E3) were evaluated. Table 1 presents the properties of the scanners. The required number of repetitions for accuracy assessment of each scanner was calculated to be 7 according to a previous study [ 11 ] assuming α=0.05 and power of 90%. Each of scanners performed 7 scans of implant abutment of SIC (SIC MAX) fixed in a typodont model of the maxilla at the site of maxillary right canine (Figure 1). 

**Table 1:** Properties of the scanners used in this study

3Shape Trios	Intraoral	Confocal	3Shape, Denmark
Sirona InEos InLab	Extraoral	Structured light	Dentsply Sirona
Sirona X5	Extraoral	Structured light (blue)	Dentsply Sirona
Dentium	Extraoral	Structured light (white)	Dentium, South Korea
imes icore 350i	Extraoral	Structured light	Imes Icore, Germany
Amann Girrbach	Extraoral	Structured light	Amann Girrbach, Austria/Germany
3Shape D700	Extraoral	Laser	3Shape, Denmark
3Shape E3	Extraoral	LED Blue	3Shape, Denmark

In order to assess the effect of presence/absence of an adjacent tooth on the accuracy of scanner, lateral incisor was present and first premolar was absent. The abutment was also scanned with 3Shape Trios intraoral scanner to serve as the reference scan for the purpose of comparison. The scanned files were converted to stereolithography (STL) format using Cross Manager 2017 software. These files included 3D coordinates of each scanned point of the abutment surface. In order to eliminate the margins, in all STL files, the area right under the abutment finish line was cut. Each scan was superimposed on the reference scan using the best-fitted mathematical algorithm and compared using 3D Geomagic Wrap 2017 software. The distance from the surface of the reference scan to each scan at different points was measured to determine the trueness and was reported qualitatively and quantitatively by the software. Geomagic Wrap 2017 3D analysis software superimposed two scans with the most fitted algorithm and analyzed their difference at 115 points in millimeters. It also calculated the maximum positive and negative discrepancies, the average positive and negative discrepancies, the mean, and standard deviation of each comparison and the root mean square (RMS). 

**Figure 2:**
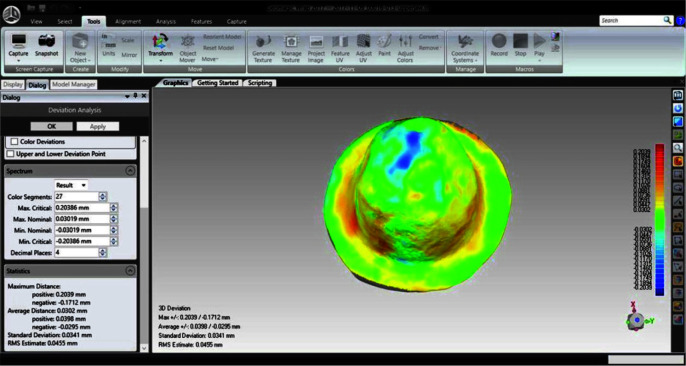
Three-dimensional comparison of scans with the reference scan using Geomagic Wrap software

Moreover, it offered a color model of the difference between the analyzed scans. A positive distance value indicated that the respective point was out of the boundaries of the reference scan and was marked in yellow. A negative value indicated adaptation and presence of point within the boundaries of the reference scan and was 

marked in green (Figure 2). The data were analyzed using SPSS version 22 (SPSS Inc., IL, USA). The trueness of each scanner was determined based on the RMS (mean of the square of the difference between the two scans at the measured points) of the seven scans in comparison with the reference scan. Precision was determined based on the mean rank of standard deviation (standard deviation of differences measured at different points) of seven scans compared with the reference scan [ 16 ]. The mean values were analyzed using the Kruskal-Wallis test and Bonferroni test.

## Results

### Trueness

Descriptive statistics regarding trueness showed that E3 scanner had the highest trueness with a mean value of 35.37 µ while X5 scanner with a mean value of 51.78 µ had the lowest trueness. Table 2 shows the mean trueness of different scanners. The Kruskal-Wallis test showed a significant difference in the mean trueness of different scanners (*p*= 0.003). Thus, pairwise comparisons were carried out (Table 3).

**Table 2:** Raw data used for statistical analysis on trueness (µm)

	Mean	Std. Deviation	Std. Error	95% Confidence Interval for Mean		Minimum	Maximum
				Lower Bound	Upper Bound		
Amann Girrbach	41.4714	2.76930	1.04670	38.9102	44.0326	38.50	45.50
Dentium	42.9286	11.72344	4.43104	32.0862	53.7709	32.60	65.50
X5	51.7857	7.67755	2.90184	44.6852	58.8863	35.60	59.50
D700	47.2714	14.37344	5.43265	33.9782	60.5646	34.90	75.00
E3	35.3714	3.42672	1.29518	32.2022	38.5406	31.20	39.70
Imes	48.6857	3.20958	1.21311	45.7173	51.6541	42.40	52.00
Ineos	48.6600	3.90743	1.74746	43.8083	53.5117	41.90	51.40

**Table 3:** Pairwise comparisons of scanners in terms of trueness (level of significance: 0.05)

Scanner	Trueness (µm), Mean	Adj. Sig. Values						
		E3	Dentium	D700	X5	Imes	Ineos	Amann Girrbach
E3	53.37	1.000	1.000	.385	.002	.048	.106	1.000
Dentium	42.92	1.000	1.000	1.000	.311	1.000	1.000	1.000
D700	47.27	.385	1.000	1.000	1.000	1.000	1.000	1.000
X5	51.78	.002	.311	1.000	1.000	1.000	1.000	.462
Imes	48.68	.048	1.000	1.000	1.000	1.000	1.000	1.000
Ineos	48.66	.106	1.000	1.000	1.000	1.000	1.000	1.000
Amann Girrbach	47.47	1.000	1.000	1.000	.462	1.000	1.000	1.000

### Precision

Descriptive statistics regarding precision showed that E3 scanner had the highest precision with a mean value of 35.34 µ. The lowest precision was noted in 3Shape D700. Table 4 shows the mean precision of different scanners.

**Table 4:** Raw data used for statistical analysis on precision (µm)

	Mean	Std. Deviation	Std. Error	95% Confidence Interval for Mean		Minimum	Maximum
				Lower Bound	Upper Bound		
Amann Girrbach	36.9143	1.98530	.75037	35.0782	38.7504	34.10	39.70
Dentium	42.2571	11.66574	4.40924	31.4681	53.0462	31.60	64.50
X5	47.1714	7.50593	2.83698	40.2296	54.1133	32.20	55.00
D700	47.2286	14.30416	5.40647	33.9994	60.4577	34.90	74.80
E3	35.3429	3.46403	1.30928	32.1392	38.5466	31.20	39.70
Imes	46.3571	3.70399	1.39998	42.9315	49.7828	38.90	50.10
Ineos	44.9400	4.50699	2.01559	39.3438	50.5362	37.40	48.50

The Kruskal-Wallis test showed a significant difference in the mean precision of different scanners (*p*= 0.014). Thus, pairwise comparisons were carried out (Table 5).

**Table 5:** Pairwise comparisons of scanners in terms of precision (level of significance: 0.05)

Scanner	Precision (µm), Mean	Adj. Sig. Values						
		E3	Dentium	D700	X5	Imes	Ineos	Amann Girrbach
E3	35.34	1.000	1.000	.416	.066	.080	.529	1.000
Dentium	42.25	1.000	1.000	1.000	1.000	1.000	1.000	1.000
D700	47.22	.416	1.000	1.000	1.000	1.000	1.000	1.000
X5	47.17	.066	1.000	1.000	1.000	1.000	1.000	.337
Imes	46.35	.080	1.000	1.000	1.000	1.000	1.000	.395
Ineos	44.94	.529	1.000	1.000	1.000	1.000	1.000	1.000
Amann Girrbach	36.91	1.000	1.000	1.000	.337	.395	1.000	1.000

## Discussion

The results showed a significant difference in precision and trueness of different scanners. Thus, the null hypotheses of the study were rejected. In this study, the coordinate measuring machine was not used due to the small size of implant abutment. Instead, 3Shape Trios intraoral scanner was used as reference. Lee *et al*. [ 3 ] assessed the accuracy of scanners and used Blue Light intraoral scanner as reference. Moreover, Renne *et al*. [ 11 ] showed that 3Shape Trios scanner had the highest speed and accuracy. In addition, Vandeweghe *et al*. [ 17 ] and Nedelcu *et al*. [ 18 ] showed that 3M True Definition and Trios scanners demonstrated the highest accuracy. 

Our results revealed significant differences among scanners in terms of trueness and precision. After 3Shape Trios (reference scanner), 3Shape E3 extra oral scanner had the highest trueness with significant differences with Sirona X5 and Imes. The difference of scanners with the reference scanner was below the clinically acceptable threshold of 75 µ [ 16 ] but was higher than the rate reported by the manufacturers (between 10 to 20 µ) [ 1 ]. Our findings in this respect were in line with the results of previous studies [ 1 , 19 - 22 ]. For instance, De Villaumbrosia *et al*. [ 1 ] reported a trueness value of 38.8 µ and a precision value of 45.5 µ for structured light extra oral scanner. Persson *et al*. [ 19 ] reported a trueness of 10 µ for contact scanners. HYPERLINK "https://www.sciencedirect.com/science/article/pii/S1883195818303268" \l "!" Faruk Emir *et al*. [ 20 ] reported that scanners using blue-light showed more accurate results than the white-light and laser scanners. Del corso *et al*. [ 21 ] reported that the trueness of structured light scanners was 14 to 21µ, which was higher than the values reported by Persson *et al*. [ 19 ] DeLong *et al*. [ 22 ] used a coordinate measuring machine and reported the trueness of structured light scanners to be 18 to 30µ. In addition, the mean precision of each scanner was lower than the trueness of the respective scanner.

De Villaumbrosia *et al*. [ 1 ] reported that the mean precision of each scanner was higher than its trueness; however, they attributed this finding to the sharp edges of the fabricated dies in their study.

It should be noted that our results could not be accurately compared with the results of previous studies. We used the RMS output of the software to measure the mean trueness of each scanner, similar to some previous studies [ 16 , 23 ].

However, some other studies reported the general mean output of the software [ 24 ]. In case of using the general mean (positive and negative deviations), the trueness would be zero given that the data are normally distributed [ 25 ]. Hack and Patzelt [ 14 ] found that 3Shape Trios intraoral scanner had the highest trueness. 

In order to scan an object with extra oral scanners, the object should be coated with a specific powder due to the contrast of light, which is not required for intraoral scanners [ 3 ]. The thickness of this powder may further contribute to the difference between the tested scanners and the reference scanner.

Maximum precision was noted in E3 scanner with significant differences with X5, Sirona InLab, D700, and Imes. In addition, the difference in this respect was significant between Amann Girrbach scanner and Imes, X5 and D700 scanners. The lowest precision was noted in 3Shape D700 laser scanner; further investigations are required on this topic. Ender and Mehl [ 6 ] found that the precision of intraoral scanners decreased as the distance from the object to scanner increased. In addition, De Villaumbrosia *et al*. [ 1 ] found that Zeno Scan extraoral scanner had the highest accuracy and precision. 

The controversies in the results of these studies can be due to different methodologies and different scanners employed. Moreover, it should be noted that scanners are not the same in different parameters and one scanner may be superior to others in one parameter and inferior to other scanners in another parameter. This is also true about the technologies used in scanners [ 1 ]. For example, De Villaumbrosia *et al*. [ 1 ] found that laser scanners had the highest trueness (35.5 µ) and precision while the performance of light scanners in their study was not suitable. However, Chan *et al*. [ 9 ] reported that the performance of laser scanners was superior to that of light scanners. Jeong *et al*. [ 15 ] compared intraoral scanners and Blue Light scanners and concluded that no significant difference existed between the two. As mentioned earlier, different methodologies and use of different types of scanners may explain the difference in the results.

## Conclusion

Within the limitations of this study, the results showed that the tested scanners had significant differences with each other in terms of trueness and precision, and 3Shape E3 extra oral scanner had the highest trueness and precision. Minimum trueness was noted in Sirona X5 and minimum precision was detected in 3Shape D700. 
